# Endoscopic intervention in a case proven latterly to be a COVID-19

**DOI:** 10.1186/s43162-020-00029-6

**Published:** 2020-11-25

**Authors:** Shaimaa Elkholy, Mohamed-Naguib Wifi, Karim K. Maurice, Kerolos Youssif, Karim Mashhour, Shady N. Mashhour, Ahmed Nabil, Abeer Awad Abdellatif

**Affiliations:** 1 Internal Medicine, Hepatogastroenterology Unit, Kasr Al-Ainy School of Medicine, Cairo University, Kasr Al-Aini Street, Cairo, 11451 Egypt; 2 Department of General and Laparoscopic Surgery, Kasr Al-Ainy School of Medicine, Cairo University, Cairo, Egypt; 3 Clinical Oncology, Kasr Al-Ainy School of Medicine, Cairo University, Cairo, Egypt; 4 Department of Interventional Radiology, Kasr Al-Ainy School of Medicine, Cairo University, Cairo, Egypt; 5 Department of General Surgery, Kasr Al-Ainy School of Medicine, Cairo University, Cairo, Egypt

**Keywords:** Duodenal stent, Non-operable gastric carcinoma, COVID-19, Infection control measures, Health care workers

## Abstract

**Background:**

COVID-19 pandemic caused by severe acute respiratory syndrome coronavirus 2 (SARS-CoV-2) is responsible for the newly developed worldwide outbreak of coronavirus disease with a high rate of mortality especially among elderly and multiple co-morbid personnel. Asymptomatic COVID-19-infected patients are a well-known source of transmission of infection. The risk of exposure to respiratory secretions and/or feces is hardly avoidable during the endoscopic procedure; also, the aerosol and droplets take up to an hour disperse, so they remain a risk to staff and other patients after they leave the room; therefore, strict infectious precautions should be taken by all health care workers to limit the virus spread.

**Main body:**

We present an endoscopic trial of duodenal stent insertion in non-operable gastric carcinoma that is proven 2 days later to be a COVID-19-positive case. Fortunately, no one of the health care workers that came in contact with the case becomes infected owing to the proper infection control measures.

**Conclusion:**

We recommended that the endoscopy examination and procedures should be strictly limited to urgent cases to minimize the risk of virus infection among health care workers.

## Background

On 12 December 2019, a severe respiratory disease was recently reported in Wuhan, known as COVID-19 caused by severe acute respiratory syndrome coronavirus 2 (SARS-CoV-2) which is responsible for the worldwide outbreak pandemic of coronavirus disease [1]. Patients may present with a wide variety of symptoms from mild to severe symptoms as a severe acute respiratory infection, pneumonia, and acute respiratory distress syndrome or even may be asymptomatic [2]. The described common routes of COVID-19 infection are respiratory secretions, feces, and contaminated environmental surfaces; however, it could be transmitted also from infected asymptomatic people which makes the diagnosis challenging [3]. Due to the unavoidable risk of exposure to respiratory secretions and/or feces during the endoscopic procedure, all the medical staff in the endoscopic theater are considered at high risk of exposure to COVID-19 infection, and therefore, strict infection control measures during the COVID-19 pandemic are mandatory [4]. Patients with asymptomatic COVID-19 infection are considered a high risk of transmission of infection to staff as the infection will not be obvious before having a procedure [5]. All medical staff members who come with close contact with a confirmed COVID-19-infected case should protect themselves from acquiring the infection by applying the recommended infection control protocols including wearing personal protective equipment such as eye-protective goggles, face mask, face shield, gloves, and various gowns.

## Main text

We report a case of a 40-year-old male patient, recently discovered with non-operable obstructing gastric cancer infiltrating the duodenum subjected to palliative endoscopic duodenal stenting, who proved to have a confirmed COVID-19 infection latterly. We aimed to emphasize the importance of prophylaxis measures during endoscopic procedures in the prevention of COVID-19 infection.

**Fig. 1:**
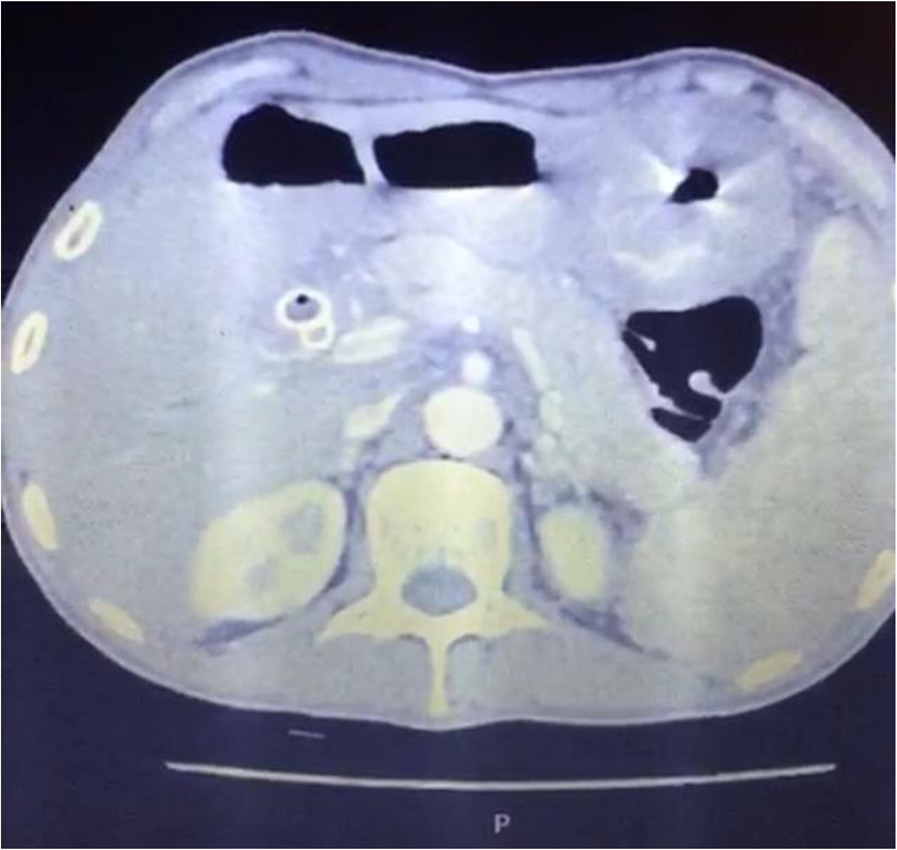
Abdominal CT revealing malignant gastric tumor

On 9 March 2020, a 40-year-old male patient complained of obstructive jaundice, vomiting, dyspepsia, and weight loss of 15 kg in the last 2 months together with a mild sore throat, but there were no fever, dyspnea, cough, or any other respiratory symptoms. The patient was not known to have diabetes, hypertension, or any other medical condition. His diagnostic upper endoscopy showed a mass in the body extending to the antrum; multiple biopsies were taken for the histopathological examination which revealed a case of moderately differentiated adenocarcinoma (T3N1M0). CT scan of the abdomen was done and revealed an obstruction to the common hepatic duct (CHD) by diffuse lymphatic permeation, rather than definite soft tissue lesions, causing dilatation of the RT and LT hepatic ducts and IHBR together with malignant duodenal infiltration causing a picture of gastric outlet obstruction (Fig. 1). Upon a multidisciplinary team (MDT) decision, the patient was subject to palliative endoscopic duodenal stenting for relieving the obstruction. On 11 March, the patient was subjected to endoscopic duodenal stenting which was done at the endoscopic theater under routine infection control measures including handwashing and using a surgical mask, disposable long-sleeve gown, and two pairs of gloves. OGD was done using a high-definition therapeutic gastroscope with an auxiliary water channel (GIF-1TH190 Olympus) with a 3.7-mm working channel; the medical team who attended the procedure was composed of the operator doctor, the assistant doctor, the anesthesiologist, and two endoscopy trained nurses. Induction with 80 mg of propofol was used for anesthesia followed by 160 mg maintenance during the duration of the procedure (45 min). The duodenal stent was tried to be inserted, but it was not feasible due to the presence of multiple levels of obstruction after the duodenum suggesting peritoneal disease despite negative CT findings so the decision was to prepare the patient for palliative surgery. During the entire procedure, there was no visible or definite exposure to any body fluids from the patient was recorded.

**Fig. 2:**
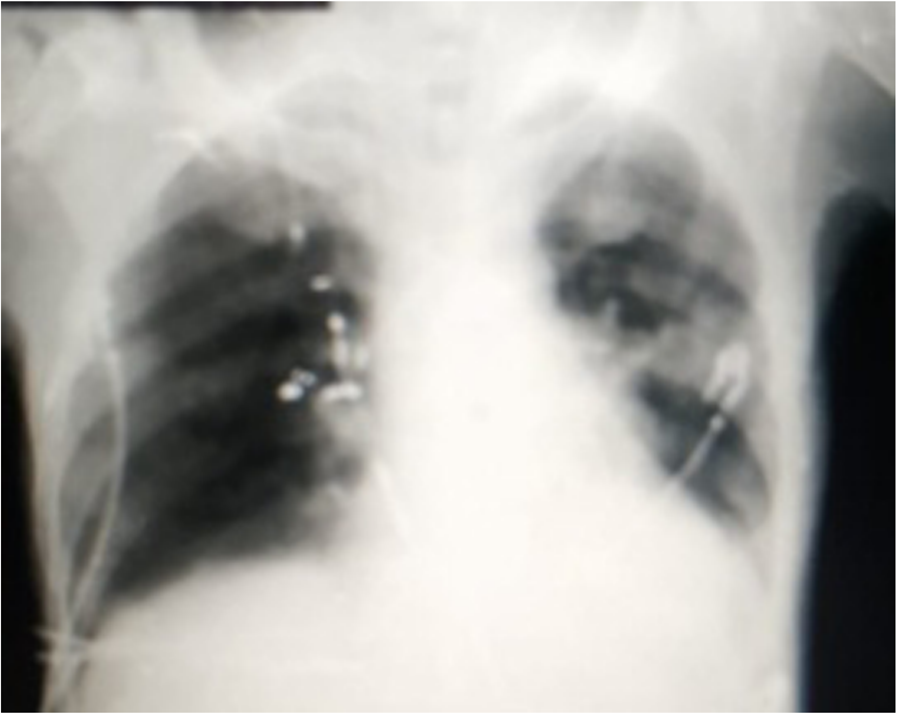
Chest X-ray (day 1) revealing bilateral ground-glass opacities more at the left lung

**Fig. 3:**
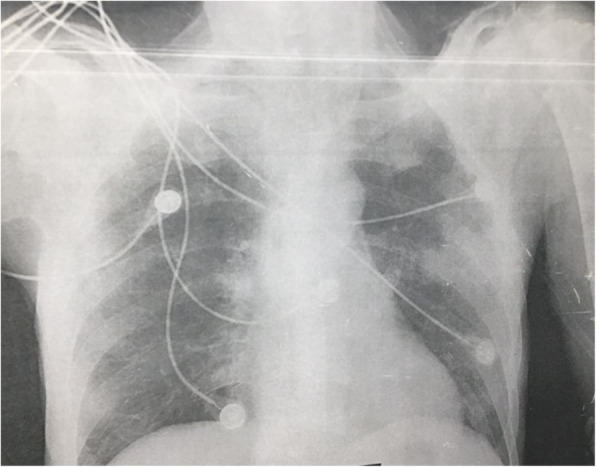
Chest X-ray (day 3) revealing multiple bilateral patchy lung consolidations and ground-glass opacities

Two days later, the patient started to develop a high-grade fever (40 °C), rapidly progressive dyspnea, hypotension, and disturbance in the conscious level, upon which he was admitted to the ICU with the diagnosis of septic shock secondary to a chest infection. On admission to ICU, the patient has a disturbed conscious level (GCS was 12 /15) with no lateralizing signs; chest examination revealed bilateral diminished air entry more to the left lung. Central venous catheter was inserted, resuscitation by IV fluids failed to achieve mean BP more than 65 mmHg so vasopressin therapy in the form of norepinephrine was started. Chest X-ray was done on day 1 of ICU admission which revealed bilateral ground-glass opacities more at the left lung (Fig. 2) upon which raise the suspicion of COVID-19 infection. Unfortunately, the patient’s condition has rapidly deteriorated with severe tachypnea (RR = 40/min), severe hypoxia (oxygen saturation was 68%), and rapidly deteriorating conscious level; therefore, the patient was intubated and mechanically ventilated. Management was started in the form of maintaining good hydration, improve oxygenation, and IV antibiotics (imipenem, vancomycin, and metronidazole). With suspicion of COVID-19 infection, an oropharyngeal swab was done by RT-PCR assay which was proven to be a COVID-19-positive infection. The patient was referred to one of the COVID-19 quarantine hospitals, but sadly, the patient’s condition has rapidly deteriorated with severe lung infiltration and development of acute respiratory distress syndrome (ARDS) (Fig. 3), and the patient died 3 days later. From a good point of view, no one from the medical staff who came in contact with the patient develops any symptoms suggestive of COVID-19 infection; also, their repeated nasopharyngeal swabs were negative.

## Conclusion

Herein, we recommend that endoscopy examination and procedures should be strictly limited to urgent cases such as gastrointestinal bleeding, acute cholangitis, biliary pancreatitis, foreign body retrieval, and selected cases of obstructive jaundice and to defer any non-urgent or elective cases. Protection of the medical staff should be the first issue to be considered through regular screening, strict infection control measures as recommended by all societies as regards the use of personal protective equipment (PPE) during the examination, and by restricting the endoscopic examination and procedures to the emergency cases only; however, there is still controversy between different societies regarding urgent procedures, postponement of non-urgent examinations, prescreening of patients, training for PPE use (to wear and to remove), contact patients 14 days after examination, and self-surveillance by HCW. It should be taken into consideration that the potential or confirmed cancer cases which are difficult to be deferred should be reviewed on a case-by-case basis.

## Data Availability

Not applicable

## Abbreviations

SARS-CoV-2: Severe acute respiratory syndrome coronavirus 2
CHD: Common hepatic duct
HCC: Hepatocellular carcinoma
MDT: Multidisciplinary team
ARDS: Acute respiratory distress syndrome
HCW: Health care worker
PPE: Personal protective equipment
